# Home Enteral Nutrition: Towards a Standard of Care

**DOI:** 10.3390/nu10081020

**Published:** 2018-08-04

**Authors:** Leah Gramlich, Ryan T. Hurt, Jennifer Jin, Manpreet S. Mundi

**Affiliations:** 1 Department of Medicine, University of Alberta, Edmonton, AB T6G 2P5, Canada; Jennifer.Jin@ualberta.ca; 2 Mayo Clinic, 200 1st Street, Rochester, MN 55905, USA; ryan.hurt@mayo.edu (R.T.H.); Mundi.Manpreet@mayo.edu (M.S.M.)

**Keywords:** home enteral nutrition, standards, enteral access devices

## Abstract

The purpose of this overview is to make the case for the establishment and publication of standards for home enteral nutrition (HEN) therapy in adult patients who require a long-term alternative to oral feeding. Overviews can provide a broad and often comprehensive summation of a topic area and, as such, have value for those coming to a subject for the first time. It will provide a broad summation, background and rationale, review specific considerations unique to HEN (tubes, products and supplies) and we describe a recent audit of seven HEN programs which highlights tube and process related challenges. Based on the overview of the literature and our experience with the audit we propose a way forward for best home enteral nutrition care.

## 1. HEN: Background and Rationale

Enteral Nutrition (EN) generally refers to nutrition therapy where liquid formula or blenderized food is delivered to the gastrointestinal (GI) tract to supplement or provide all of the caloric requirements of an individual. EN is an option for patients who are unable to meet their nutritional requirements orally but have a functional GI tract able to digest and absorb formula introduced into the lumen of the GI tract. 

EN administered to outpatients by a tube is known as home enteral nutrition (HEN) and may be an option for patients who require life-sustaining nutrition care with a long-term alternative to oral nutrition but are otherwise able to live outside of an acute care hospital facility. Some patients may be admitted to hospital for an acute or chronic illness and have enteral nutrition initiated during their hospitalization and then are discharged to the home or other community setting to continue with outpatient enteral feeding. Other patients are identified as being at nutrition risk in the outpatient setting and may be admitted specifically to initiate enteral nutrition in hospital and transition to home. In centers where there are dedicated HEN teams to provide monitoring, it is also possible to have enteral access placed and initiate feeding without requiring admission to hospital. Criteria for admission to HEN programs may vary but include a variety of considerations (Table 1). Administration of tube feeding outside of an acute care facility requires that the patient and/or their caretakers learn to administer feeds, care and maintain the enteral tube, and understand the equipment involved, including an infusion pump if applicable. In addition, they must arrange receipt of delivery of enteral nutrition formula and related supplies and ensure a sanitary living environment with clean water and electricity. 

## 2. HEN: Programs

The availability of a dedicated HEN program consisting of healthcare providers, including nurses, dietitians, physicians, speech and occupational therapists, varies between countries and centers. The grey literature includes standards for HEN in certain jurisdictions but there are no standards published in the peer-reviewed literature. An observational study by Silver et al. [1] suggested that patients who are not part of a HEN program face many challenges, including maintenance of functional status, access to an interdisciplinary team, complications of EN, and caregiver competence. With an increase in number of patients receiving HEN, the importance of support by a multidisciplinary team is increasingly being recognized [2]. Some HEN programs are fully funded by the government whereas patients in other regions may have to pay a portion or all of the costs of tube feeding in the home. Klek et al. [3] was in a unique position to report on both. Patients in Poland were previously using blenderized homemade feeds and not routinely monitored until reimbursement was introduced in 2007 where patients switched to commercial EN products and followed by a HEN support program. Following implementation of these changes, there was a reduction the incidence of infectious complications, number of hospital admissions, and length and costs of hospital stay. It was not possible to attribute the changes to either the effect of the change to a commercial formula versus the addition of complex care from a specialized HEN team. 

## 3. HEN: Patients/Clients

HEN patients are a heterogeneous group. Collectively, patients receiving HEN have significant comorbidities and in addition to challenges related to getting nutrients in, they routinely also have major difficulties in other activities of daily living such as mobilization, cognition and elimination. In 1999, a multicenter retrospective survey of HEN in Europe was performed to assess its practice in 23 centers in 8 different countries [4] and involved adult patients that were newly enrolled in the respective HEN programs the full year prior. A total of 1397 patients were registered with about 55% of patients being older than 65 years, and 21% over 80 years. The main reason for requiring HEN was dysphagia (84.6%) and the underlying diseases were a neurological disorder (49.1%), or head and neck cancer (26.5%). In this study the majority of patients were fed by a percutaneous endoscopic gastrostomy (PEG) (58.2%) or a nasogastric tube (29.3%) and most infusions were cyclical (61.5%) rather than bolus (34.1%). In another retrospective cohort study of 727 adults admitted to a comprehensive, publicly funded HEN program where all patients had feeding via PEG tube, 46% of patients had cancer (largely head and neck and esophagus), 32% had neurologic disease (neurovascular or neurodegenerative) and 15% had gastrointestinal diseases [5]. 

Selection of patients for HEN is important given that it is often care provided at end of life and for advanced disease with inability to swallow. In addition to ethical considerations of non-volitional feeding, there is little evidence of benefit in HEN in patients with advanced dementia. The European Society of Enteral and Parenteral Nutrition guidelines on nutrition in dementia recommends against the initiation of enteral nutrition in patients with advanced dementia [6]. A Cochrane review from 2009 found no evidence of increased survival in advanced dementia patients being tube fed [7] but there also are studies showing there may be some harm [8]. 

It is important to also consider the caregivers for HEN patients. Feedback from caregivers in a HEN center in Italy showed that they valued home visits for clinical follow-up but also for providing psychological support [9]. While they may feel socially isolated and psychologically burdened from the additional roles they take on, overall feedback from caregivers was positive and having their family member at home was important. 

## 4. HEN: Prevalence

The annual prevalence of HEN is dramatically increasing in the US with an increase in prevalence from 463 per million population in the United States in 1995 [10] to 1385 per million in a recent report [11]. A 2011 report from the UK showed a point prevalence of 92 per million population with 71% of the patient population continuing on HEN in the following year [12]. A European multicenter survey from 2003 found the median incidence of HEN from a variety of centers to be 163 per million population per year with a range of 62–457 [4]. In a study of all patients in Northern Alberta, Canada on HEN, the incidence was 150/million population [5].

## 5. HEN: Impact on Outcome

While there are large numbers of patients utilizing this life sustaining therapy, cost utilization and economic impact and outcomes of HEN are not clear. Both the underlying indication for HEN and the provision of HEN itself impact the outcome of patients on HEN. In a study that looked at outcome on HEN relative to indications it was noted that patients who start on HEN have high mortality rates with death accounting for over 50% of all discharges in patients with both cancer and neurological indications for HEN [5]. Patients with neurological disorders remained on HEN program significantly longer than patients with cancer as patients with cancer are more likely to transition to oral intake. At the end of one year, approximately 25% of patients with neurological disease were still in the program compared with 15% of cancer patients. Patients receiving HEN have significant comorbidities challenges and quality of life and cost-effectiveness of therapy are important to consider. A recently published systematic review on the health economic implications of HEN in individuals living at home and long-term care/institutionalized patients concluded that there is a paucity of good quality economic studies [13]. The overall cost for a HEN patient was generally about $10,000 to $20,000 US per year. HEN therapy was found to be cost effective in certain groups for prolonged survival. Elia et al. [14] performed a cost analysis in stroke patients receiving tube feeding in the home or nursing home and found that HEN was cost-effective in the UK when non-medical costs were paid privately. In terms of other clinical outcomes related to HEN, Hisashige [15] performed a small randomized control trial to provide high protein/high calorie tube feeding to immobile patients with pressure ulcers versus standard calorie and protein formula. The nutritional intervention reduced the number of pressure ulcer days, increased quality-adjusted life years, and reduced costs per person overall. A HEN center in France studied their patients who mostly had neurological indications for EN and found that quality life was poorer in comparison to the general population although results were sometimes better in younger patients, patients without cancer, and patients with more than one care-give [16]. In addition, most patients reported improved or stable mental and physical well-being since enrollment in HEN program. 

## 6. Practical Considerations in Home Enteral Nutrition

### 6.1. Enteral Access Devices

When considering enteral nutrition, there are multiple options for obtaining access to the stomach or intestines. Naso-enteric tubes (NET) refer to any feeding tube that is placed nasally into the esophagus and beyond. They are further named based on the distal most location of the tube; nasogastric (NG) for tip in the stomach, nasoduodenal (ND) for tip in the duodenum, and nasojejunal (NJ) for tip in the jejunum. NG tubes have the advantage of being placed at the bedside by nursing staff and also can be used for suction or decompression. However, it is important to note that the typical feeding tube has a much smaller bore size than tubes used for decompression in an effort to increase patient tolerance and comfort. Typically, NET are recommended for short term use only (<6 weeks) as prolonged use of NET can lead to complications including lesions/erosions of the nasal wing, chronic sinusitis, gastro-esophageal reflux, and possible aspiration pneumonia [17,18]. The use of NET in HEN has not been reported upon in the literature but in practice it is being used pre-operatively in patients with esophageal cancer who are malnourished and in whom the use of a percutaneous tube for enteral access may have negative impacts on planned surgery.

For long-term HEN, enteric tubes are inserted directly either surgically or with guidance from fluoroscopy (interventional radiology) or endoscopy (gastroenterology). The surgical placement of gastrostomy was first successfully carried out in 1876 with Stamm devising the technique used most frequently today [18,19]. Technique of percutaneous endoscopic gastrostomy (PEG) or insertion of gastrostomy tube percutaneously under endoscopic guidance was introduced in 1980 by Gauderer allowing for access to the stomach without laparotomy [20]. The determination of tip location on the other hand is dependent on evaluation of patient’s clinical status. Gastric tubes require that there are no significant barriers to gastric feedings such as gastroparesis, gastric outlet obstructions, or significant reflux/hiatal hernia. In those situations, jejunal or small bowel feeding is often preferred. 

In terms of placement and choice of tubes, a number of key studies have compared each of the above approaches. When evaluating the use of NET versus percutaneous tubes, a recent Cochrane review provides guidance as it compared the use of PEG versus NG in adults with dysphagia arising from any cause [18]. They noted that PEG was associated with lower risk of treatment failure consisting of feeding interruption, blocking or leakage of the tube, and non-adherence (risk ratio (RR) 0.18; 95% confidence interval (CI) of 0.05 to 0.59). There was not significant difference in mortality (RR 0.86; 95% CI of 0.58 to 1.28) or pneumonia (RR 0.70; 95% CI 0.46 to 1.06). They did find that meta-analysis favored PEG placement for quality of life measures including inconvenience (RR 0.03; 95% CI 0.00 to 0.29), discomfort (RR 0.03; 95% CI 0.00 to 0.29), altered body image (RR 0.01; 95% CI 0.00 to 0.18; *p* = 0.001) and social activities (RR 0.01; 95% CI 0.00 to 0.18). Due to these findings, NET use should only be used in patients who require short-term (less than 4 weeks) enteral feeds [21]. Additionally, placement often arises as a barrier to NET use as most care facilities will not accept patients with NETs due to high failure rate, however, some programs use NET more frequently.

Similarly, many studies have compared the placement of percutaneous enteric tubes with endoscopic versus fluoroscopic guidance. One large trial in 378 patients (268 endoscopically placed and 110 fluoroscopically placed) noted that overall complication rate was 35% with 30% for endoscopic placement and 70% for fluoroscopic placement [22]. Malposition and leaks were higher in the fluoroscopic placement and contributed to the overall higher complication rate. Another trial also revealed similar results in 370 tubes (177 endoscopically placed and 193 fluoroscopically placed) with fluoroscopic placement having higher complication rates (23% versus 11%, *p* = 0.002) [23]. Other studies have found no major differences or advantages to fluoroscopic placement such as the requirement of less conscious sedation [24,25]. Despite these findings, the approach to placement and the team placing enteric tubes is often dependent on the expertise available in the local center. There are no studies in the literature related to routine changes of tubes.

### 6.2. Tube Complications

Tube clogs are a common complication and tend to occur due to a number of reasons including small bore of feeding tubes, slow administration rate of EN, accumulation of formula sediment in the lower segment of the tube, as well as improper administration of mediations through the tube [21,26]. Water flushes of 30 mL every 4 h during continuous feeds and before and after bolus feedings or medication administration are recommended in order to reduce clogging [21,27,28]. The volume of flushes may need to be adjusted based on clinical needs of the patient. In addition to water, many other solutions are used by patients in an attempt to unclog the tube. Marcuard et al., evaluated 6 solutions including meat tenderizer, cola and other carbonated beverages and pancreatic enzymes for their ability to unclog clotted enteral products [27]. Distilled water was used as the control and they noted that best dissolution occurred with pancrealipase in pH 7.9 solution. In a follow up study they noted that activated pancreatic enzyme was successful in clearing obstruction 72% of the time in cases [26]. 

Peristomal infections are another common complication associated with percutaneous tubes with reported rates as high as 5%–30% with high risk patients being those with type 2 diabetes, advanced malignancy, colonization with methicillin-resistant *Staphylococcus aureus*, or severe malnutrition [29]. The prevalence rates continue to be variable due to lack of objective criteria for diagnosis. We recently evaluated the use of objective criteria to stratify and treat patients who presented with complaints consistent with infection. Variables evaluated included erythema, induration, and exudates (Table 2) with a total score of 8 or greater being considered positive and treated with course of infections. Patients were subsequently followed prospectively for treatment response and/or progression of infection. We noted that 37.2% had a score of 7 or less. Out of this group, only two required further treatment. 37.2% had a score of 8 or 9 and responded to antibiotic treatment. The remaining 25.6% had a score of 10 or higher with 45% being admitted subsequently for inpatient treatment. Based on this scoring system, patients are able to be stratified into low-risk, moderate-risk, and high-risk, allowing for closer follow up of select patients and avoidance of unnecessary antibiotics in others. 

Given HEN is provided over a long period of time, a strategy is required for regular tube changes and for supporting patients in tube care as it relates to optimizing care of tubes as well as minimizing and treating complications related to their enteral access devices.

## 7. Nutritional Adequacy: Assessment, Products, Care

### 7.1. Assessment 

To date, standards for assessment and follow up of patients on HEN have not been articulated by organizations such as the American Society for Parenteral and Enteral Nutrition (ASPEN) and the European Society for Nutrition and Metabolism (ESPEN). Within formal HEN programs, standards are typically identified and include regular review with weight measurement, review of symptoms (including vomiting, aspiration, bowel movement frequency, skin issues) tube feeding tolerance and issues with tube care. The role of anthropometry to assess body composition in this patient population has not been studied.

### 7.2. Enteral Nutrition Products

In addition to selection of type of tube, the enteral nutrition formula can also be a difficult decision as the number of commercially prepared enteral products has increased significantly since their introduction in the 1940s [21]. It is important to note that the Food and Drug Administration does not regulate enteral products as they are considered a medical food, that is, a food which is formulated to be consumed or administered enterally under the supervision of a physician and which is intended for the specific dietary management of a disease or condition for which distinctive nutritional requirements, based on recognized scientific principles, are established by medical evaluation [21]. This point is important because the accuracy of the enteral formula labeling thus is dependent on the formula vendors. 

The choice of enteral formula usually starts with establishing the caloric and protein needs of the patient. Caloric requirements can be calculated with the use of a number of equations (Harris Benedict equation) as well as indirect calorimetry in hospitalized patients but typically fall in the range of 25–30 kcal/kg/day [30,31]. Similarly, protein requirements can range from 0.8–1.2 g/kg/day in most home nutrition patients. Although beyond the scope of this review, clinical conditions such as obesity, renal insufficiency, malignancy, metabolic abnormalities, etc. can affect the calorie and protein requirement and adjustments need to be made to compensate for the clinical condition of the patient.

A standard polymeric enteral formula can meet the needs of most patients and has been the recommended formula to start with even in hospitalized or critically ill patients [32]. Higher concentration may be needed depending on the fluid needs of the patient. Provision of enteral formula can occur in multiple ways. Gastric feeding can be provided in an intermittent or bolus manner with EN being delivered through either a gravity bag or syringe feeding over 15–30 min. To assist in developing tolerance, patients are often started with a feeding of 125 mL or 1/2 of a can per feeding and then then titrated up as tolerated by the patient until they are providing goal feedings. Patients are also encouraged to remain in a sitting or semi-recumbent position with head of bed (HOB) >30 degrees as data in critically ill patients reveals greater reflux and aspiration of gastric contents in patients with HOB <30 degrees [33]. Jejunal/small bowel feedings on the other hand require the use of a pump. They are typically started at 10–20 mL/h and titrated up with an eventual reduction in the duration of feedings. Typically, patients are able to tolerate feedings of 100–120 mL/h over 12 h and provide those feedings overnight. Supplements to tube feeding may include fiber, protein, fluid or additional vitamins or minerals.

After initiation of feeds, patients should be contacted to ensure they are tolerating the EN program well. Typically, symptoms of intolerance include increased fullness, gas/bloating, reflux, diarrhea or constipation. Each of these symptoms does need to be evaluated with the patient’s clinical status in mind, however some general changes could be considered. As an example, in a patient with constipation, if clinically feasible, increasing water flushes or adding fiber to the formula may be options to consider. At times, the rate of feedings may also be leading to intolerance. As opposed to eating, where the chewing process limits the rate of delivery to the stomach, with EN patients can provide feeds very quickly especially with syringe or gravity feeds. Therefore, one of the first changes to consider may be to decrease the rate of feeds and ensure that patients are taking the appropriate amount of time providing syringe or gravity feeds. If this is not successful in improving symptoms, consideration should be made to use an alternative formula such as a semi-elemental formula or blenderized tube feeding (BTF). BTF use has been increasing recently as more patients desire whole foods, organic ingredients, and consumption of foods/meals the family is eating [26,34,35].

Complications related to feeding include failure to thrive, tube feeding intolerance, constipation, reflux and regurgitation. In addition, many patients who are on HEN are candidates for transition to oral feeding. In HEN patients, a multidisciplinary team consisting of a physician, nurse, dietitian, speech-language pathologist and or occupational therapist can work with the patient and their caregiver to optimize care through ongoing assessment and intervention.

## 8. HEN Benchmarking

To date, unlike the situation in Home Parenteral Nutrition (HPN), there is a paucity of data on programmatic care and expected outcomes on HEN. In 2012, the Mayo Clinic undertook a benchmarking exercise with seven healthcare organizations, including those of the authors, to address the issue of developing, piloting and diffusing a standardized and integrated best practice model for adult patients requiring long-term feeding tubes and HEN. Short phone interviews and or email exchange was initiated with 20 organizations from which these seven sites were chosen to participate in the exercise. The patients included by programs approached in this quality improvement exercise shared complex medical and surgical histories, a wide variety of feeding tubes placed by a variety of services and a desire for clarity regarding follow up. The focus of the benchmarking study included four key areas: Tube selection and management, education, follow up or ongoing management of the patient and operations. The goal of the exercise was to provide recommendations and to develop a standardized and integrated best practice model for adult patients requiring long term feeding to positively impact outcomes for patients and healthcare providers. 

The results of this benchmarking exercise identified seven sites which managed over 4000 tubes for HEN patients annually. The majority of programs surveyed had standard protocols for short vs. long term feeding with a four-week cut off for permanent tubes (vs. NET) and inclusion in the program. All programs had capacity to get tube placement in a variety of locations (intragastric and post-pyloric). Gastroenterology placed the majority of tubes, followed by interventional radiology (IR). Issues that were discussed with the programs included patient related issues such as discussions regarding goals of care, follow up and optimization of symptoms, tube related care issues such as standard protocols and processes for tube replacements, tube site care, and managing tube complications such as blockage or leak, product related queries such as the adoption of a standard formulary for HEN, the inclusion of swallow assessment standards. Questions were asked related to patient and provider education and teaching. Issues related to program management and operations including use of an electronic medical record and funding were also queried.

Across the seven programs, it was identified that tube feeding patients had increased numbers in the preceding two years. Patients with tubes placed by gastroenterology, in contrast to those placed in IR, were more likely to be seen in follow up. Scheduled follow up occurred at 4/7 programs and 4/7 sites had a multidisciplinary clinic to manage patients. Only one had an integrated Information Technology (IT) solution regularly capturing and monitoring metrics on adult tube feeding patients and the majority felt this would be of value. In HEN care, there was variable accountability for the continuum of care across programs and it was observed that the processes were held together by “champions and heroes”. The need for thorough follow-up measures and/or clear instructions to referring clinicians was accomplished more readily where stand-alone clinics or HEN programs/teams existed.

Although this exercise did not identify an overall best practice, several best practice processes were illuminated including access to multidisciplinary, patient centered, team-based care for patients requiring HEN (Table 3). Coordination and follow up for enteral access devices and nutrition care require focused attention. Strategies to support patients and their families at the time of discharge and while at home on HEN including education, training and access to expertise and advice is important. System level supports for HEN include the development of processes for care such as discussions with patients or their families regarding goals of therapy and patient preferences, protocols for tube placement and replacement and site care, enteral product procurement and use, and electronic data capture to support audit of practice were also felt to be relevant (Table 2).

## 9. Perspective: Making the Case for a Standardized Approach to HEN

There are standards for HEN in certain jurisdictions which are available online [36]. These guidelines are not available in the peer reviewed published literature. These standards are well developed and focus on nutrition aspects of care with relatively little direction provided relative to enteral access and its complications. ESPEN has recently published guidelines for patients with Chronic Intestinal Failure (CIF) detailing comprehensive recommendations for safe and effective management of adult patients on home parenteral nutrition [37]. The principles of a safe and effective HPN program for CIF include provision of consistent care standards, equity of access for patients to high quality and clinically safe services, patient centered care with use resources appropriately and effectively. Audit of practice and outcome reporting to demonstrate value for money in the provision of HPN has also been recommended. The guidelines specify the goal of provision of evidence-based therapy, prevention of HPN related complications such as catheter-related infections and metabolic complications and ensure quality of life is maximized. In contrast to HEN and because of its incremental cost and complexity, HPN is most commonly provided in the context of multidisciplinary team based care that is funded. HEN is similar to HPN in that it reflects the provision of life-sustaining nutrition care to clients in the home, through a feeding device and with nutrients or foods that have been modified for provision through the tube. In addition, both HEN and HPN require significant involvement of the patient and or their family member or supports in the community.

The development of the ESPEN HPN guidelines provides an impetus to consider a similar approach in HEN where a model of integrated multidisciplinary care can be benchmarked to an evidence-based standard. In HEN, such a standard should address both processes of care and specific care elements related to patient oriented care considerations (such as training and education, care planning), enteral access device considerations such as insertion and replacement strategies and strategies to deal with complications, nutrition care considerations including mechanism of feeding, enteral product composition, use of supplements (fiber, protein, vitamins and minerals) and provision of supplies. Funding models and strategies to address gaps in funding should also be identified. In addition, ongoing attention must be paid to the impact of such care on outcomes such as cost, safety, quality of life and caregiver quality of life. The inclusion of quality improvement strategies such as audit of practice supports ongoing reassessment and addresses the value of HEN. There is surprisingly little evidence related to care of patients on HEN given their complexity and the cost of their care. Increasing awareness of this growing population across the developed world is an opportunity to advance research in this field. Through the development and standardization of evidence based approaches to HEN we position ourselves to advocate for HEN care for patients and providers and in doing so, to optimize care in this patient population.

## Figures and Tables

**Table 1:** Admission criteria for home enteral nutrition (HEN).

Admission Criteria for Home Enteral Nutrition
o Inability to meet nutrition requirements orally o Requirement of supplement feedings via an enteral access device o Estimated duration of therapy a minimum of 4 weeks at home o Stable clinical status o Ability to demonstrate tolerance of the treatment regimen o Patient and family acceptance o appropriate and safe home environment

**Table 2:** Peristomal infection.

Erythema
o 0 = no redness o 1 = 0 < R ^1^ ≤ 5 mm o 2 = 5 mm < R ≤ 10 mm o 3 = 10 mm < R ≤ 15 mm o 4 = 15 mm < R
Induration
o 0 = no induration o 1 = 0 < I ^2^ ≤ 5 mm o 2 = 5 mm < I ≤ 10 mm o 3 = 10 mm < I ≤ 20 mm o 4 = induration greater than 20 mm
Exudates
o 0 = no exudates o 1 = serous o 2 = serosanguinous o 3 = sanguinous o 4 = purulent

^1^ R means redness. ^2^ I means induration.

**Table 3:** Standardized HEN Program Components.

Program Component	Processes
Patient Oriented Care	Goals of Care Consent Admission Criteria Patient and family involvement Education for patients and families
Enteral Access	Tube Insertion (gastrointestinal (GI), Diagnostic Imaging) Short term vs. Long term tubes Gastric vs. Jejunal tubes Tube Replacement Tube “formulary” Site Care Tube Complications (infection, blockage, inadvertent removal) Supplies
Nutrition Care	Assessment Enteral Formulary Supplemental products Product Delivery
Swallow Evaluation	Oral Care Transition to Oral Intake
Interdisciplinary Team	Physicians (GI, Other), Nursing, Dietitian, Occupational Therapy/Speech Language Pathology, Diagnostic Imaging
Continuum of Care	Discharge Planning Follow-up Communication Links to primary care, homecare, long-term care
Operational Support	Data capture: Electronic Medical Record supports Funding/Coverage for HEN Communication Measurement Quality Improvement
